# Direct differentiation of bone marrow mononucleated cells into insulin producing cells using pancreatic β-cell-derived components

**DOI:** 10.1038/s41598-019-41823-9

**Published:** 2019-03-29

**Authors:** Ju Eun Oh, Ok Kyung Choi, Ho Seon Park, Hye Seung Jung, Su Jeong Ryu, Yong Deok Lee, Seung-Ah Lee, Sung Soo Chung, Eun Young Choi, Dong-Sup Lee, Yong Song Gho, Hakmo Lee, Kyong Soo Park

**Affiliations:** 1 Biomedical Research Institute, Seoul National University Hospital, Seoul, 03080 Republic of Korea; 2 Department of Molecular Medicine and Biopharmaceutical Sciences, Graduate School of Convergence Science and Technology, Seoul National University, Seoul, 03080 Republic of Korea; 3 Department of Biomedical Sciences, Seoul National University College of Medicine, Seoul, 03080 Republic of Korea; 4 Department of Internal Medicine, Seoul National University College of Medicine, Seoul, 03080 Republic of Korea; 5 Department of Life Sciences, Pohang University of Science and Technology, Pohang, Gyeongbuk 37673 Republic of Korea; 6 Veterans Medical Research Institute, Veterans Health Service Medical Center, Seoul, 05368 Republic of Korea

## Abstract

Transplantation of stem cell-derived insulin producing cells (IPCs) has been proposed as an alternative to islet transplantation for the treatment of diabetes mellitus. However, current IPC differentiation protocols are focused on generating functional cells from the pluripotent stem cells and tend to rely on multistep, long-term exposure to various exogenous factors. In this study, we addressed the observation that under stress, pancreatic β-cells release essential components that direct the differentiation of the bone marrow nucleated cells (BMNCs) into IPCs. Without any supplementation with known differentiation-inducing factors, IPCs can be generated from BMNCs by *in vitro* priming for 6 days with conditioned media (CM) from the β-cells. *In vitro* primed BMNCs expressed the β-cell-specific transcription factors, as well as insulin, and improved hyperglycemia and glucose intolerance after transplantation into the streptozotocin-induced diabetic mice. Furthermore, we have found that components of the CM which trigger the differentiation were enclosed by or integrated into micro particles (MPs), rather than being secreted as soluble factors. Identification of these differentiation-directing factors might enable us to develop novel technologies required for the production of clinically applicable IPCs.

## Introduction

Diabetes mellitus (DM) is characterized by chronic hyperglycemia resulting from the defects in insulin secretion, insulin action, or both. Type 1 DM results from autoimmune destruction of the β-cells in the pancreatic islets^1,2^, whereas more common type 2 DM results from insulin resistance in the peripheral tissues and subsequent β-cell dysfunction^3–5^. Although islet transplantation can achieve better glycemic control than insulin therapy^6,7^, many complicated issues including shortage of islet donors and necessity of immune suppression, have hampered this treatment’s widespread use.

During the last several decades, extensive research has been focused on the treatment of type 1 DM based on the generation of the surrogate insulin producing cells (IPCs) from the stem cells. Many research groups have developed stepwise differentiation protocols that mimic the developmental paradigms to differentiate the pluripotent stem cells (PSC) into the IPC progenitors that are capable of maturation *in vivo*^8–13^. However, IPC-generating protocols involve multistep exposure to exogenous soluble factors and/or small molecules for extended periods of time. Moreover, PSC-derived IPCs are not free from tumorigenesis after transplantation^14^. For these reasons, adult stem cells might be the preferred cell source for IPCs. Innovative differentiation technologies, which can effectively, efficiently, and reproducibly direct cell fate change, must be developed for clinical application in the diabetic cell therapy.

Extracellular vesicles (EVs) are generally referred to as exosomes, microvesicles, or microparticles (MPs), and have been implicated in numerous aspects of intercellular communication^15–19^. Recently, the pathophysiological roles of EVs are beginning to be recognized^20–23^. Moreover, it is worth noting that the EV-mediated transfer of the cellular components shed from the damaged tissues can reprogram the stem cells to acquire phenotypic features of the injured tissue cells^24–26^.

In this study, we report that bone marrow nucleated cells (BMNCs) can be differentiated into IPCs that can improve hyperglycemia in the streptozotocin (STZ)-induced diabetic mice after systemic infusion. *In vitro* priming with conditioned media (CM) prepared from the culture supernatants of the syngeneic or xenogeneic β-cells under stress conditions can direct the BMNCs to express the β-cell-specific proteins, including insulin, C-peptide, PDX-1, MafA, and Nkx6.1, within 6 days. Moreover, *ex vivo* primed BMNCs improved hyperglycemia and glucose intolerance after systemic infusion in the diabetic mice. We also found that IPC differentiation was specifically mediated by the MPs shed from the β-cells maintained under stress conditions because priming with MP-depleted CM did not induce IPC generation. These results suggest that identification of the MP-associated differentiation-directing factors might enable us to establish novel technologies for the production of IPCs.

## Results

### *In vivo* differentiation of BMNCs into IPCs

**Figure 1:**
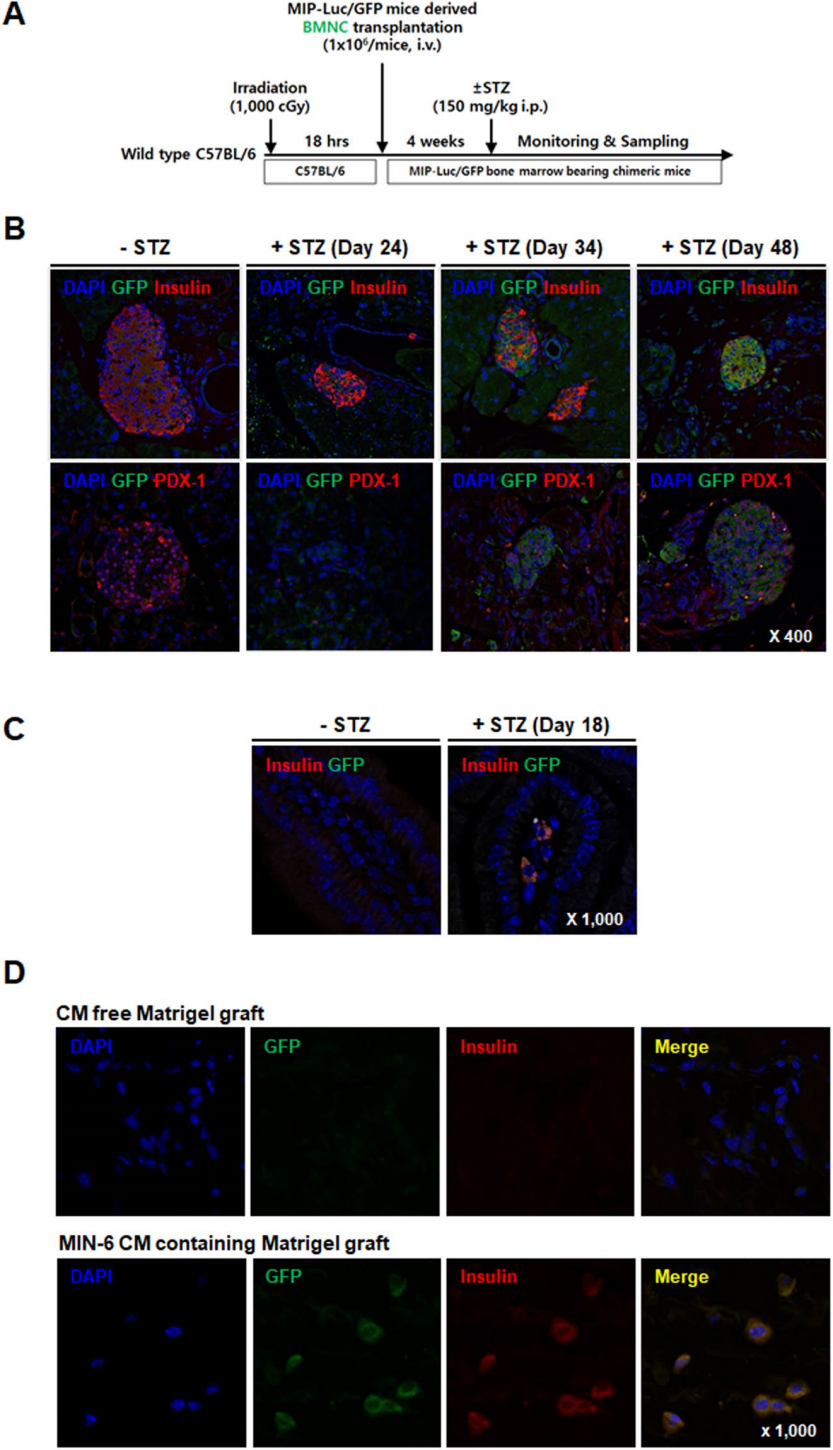
BMNCs differentiate into IPCs *in vivo*. (**A**) Outline of the experimental procedure to test the contribution of BMNCs in the generation of IPCs in STZ-induced diabetic mice. (**B**) Immunofluorescent staining of pancreatic tissues harvested from chimeric C57BL/6 mice bearing BMNCs from MIP-Luc/GFP mice. Each vertical panel show representative staining of the pancreas harvested from control (−STZ) and STZ- treated mice (24, 34 and 48 days after STZ injection, +STZ). GFP-expressing cells are present in some islets and almost all of the GFP expressing cells are stained for insulin (yellow, upper right) and PDX-1 (lower right). There are no GFP expressing cells in the islets of normal chimeric mice. Magnification 400×. Immunofluorescent staining for DAPI (blue) and GFP (green) with insulin (red) or PDX-1 (red) in pancreatic tissues. Additional single color images are provided in Supplementary Fig. S1A. (**C**) Representative immunofluorescent staining of small intestinal tissues in control (vehicle treated, −STZ) or diabetic-MIP-Luc/GFP mice (STZ treated, +STZ) on day 18. Note the appearance of insulin and GFP double positive cells in the lamina propria of the intestinal villi of diabetic mice and the absence of these cells in control mice. DAPI, GFP, and insulin staining are shown in blue, green, and red respectively. Magnification 1,000×. Additional single color images are provided in Supplementary Fig. S1B. (**D**) Representative immunofluorescent staining of a Matrigel graft harvested 18 days after transplantation as described in the supplementary methods. GFP (green) and insulin (red) double positive cells are present in the Matrigel graft containing CM of β-cells (lower panel) but not in the CM-free graft (upper panel). Magnification 1,000×. All images were acquired with an Olympus FluoView FV1000 confocal microscope.

It has been previously reported that BMNCs significantly contribute to adult β-cell renewal in mice^27–31^, but other reports have contradicted these findings^32,33^. Initially, we tested whether BMNCs can differentiate into IPCs. We generated chimeric C57BL/6 mice harboring BMNCs from the insulin promoter luciferase/GFP transgenic (MIP-Luc/GFP) mice and then treated the mice with streptozotocin (STZ) to destroy the β-cells, while control mice were treated with the same volume of vehicle (Fig. 1A). We then analyzed pancreatic sections by immunofluorescence staining with antibodies against GFP, insulin, and PDX-1 at different time points. It should be noted that the GFP-expressing cells began to appear approximately 24 days after STZ treatment, and the number of the GFP-positive cells increased up to 48 days after STZ treatment (Figs 1B; S1A; Table S1). These results implied that the GFP and insulin double positive cells were differentiated from BMNCs that were mobilized from the bone marrow. We also detected the GFP and insulin double positive cells in the small intestine on day 18 in MIP-Luc/GFP mice treated with STZ (Figs 1C; S1B), consistent with an increase in the luciferase signal in the intestine of these mice (Fig. S1C). These phenomena are similar to previous reports that have demonstrated heterotopic neogenesis of IPCs in diabetic animal models, such as STZ-treated mice^34–36^. We hypothesized that damaged β-cells might shed some factors that direct the differentiation of BMNCs into IPCs. Thus, we prepared conditioned media (CM) from the culture supernatant of an insulinoma cell line maintained under stress at low levels of glucose and serum. We mixed the CM with Matrigel at a ratio of 1:1 and transplanted the mixture into the subcutaneous region of the healthy chimeric MIP-Luc/GFP mice. Immunofluorescence staining of the Matrigel platforms harvested on day 18 after transplantation revealed newly differentiated insulin and GFP double positive cells only in the Matrigel platforms containing CM of syngeneic MIN-6 insulinoma cells (Fig. 1D). However, the CM-free Matrigel or the Matrigel platforms containing CM of a clonal endothelial bEND.3 cell line did not show any positive cells (Fig. S1D). These results indicate that BMNCs were capable of differentiating into IPCs in response to the signals or components shed from the damaged β-cells.

### *In vitro* differentiation of BMNCs into IPCs

**Figure 2:**
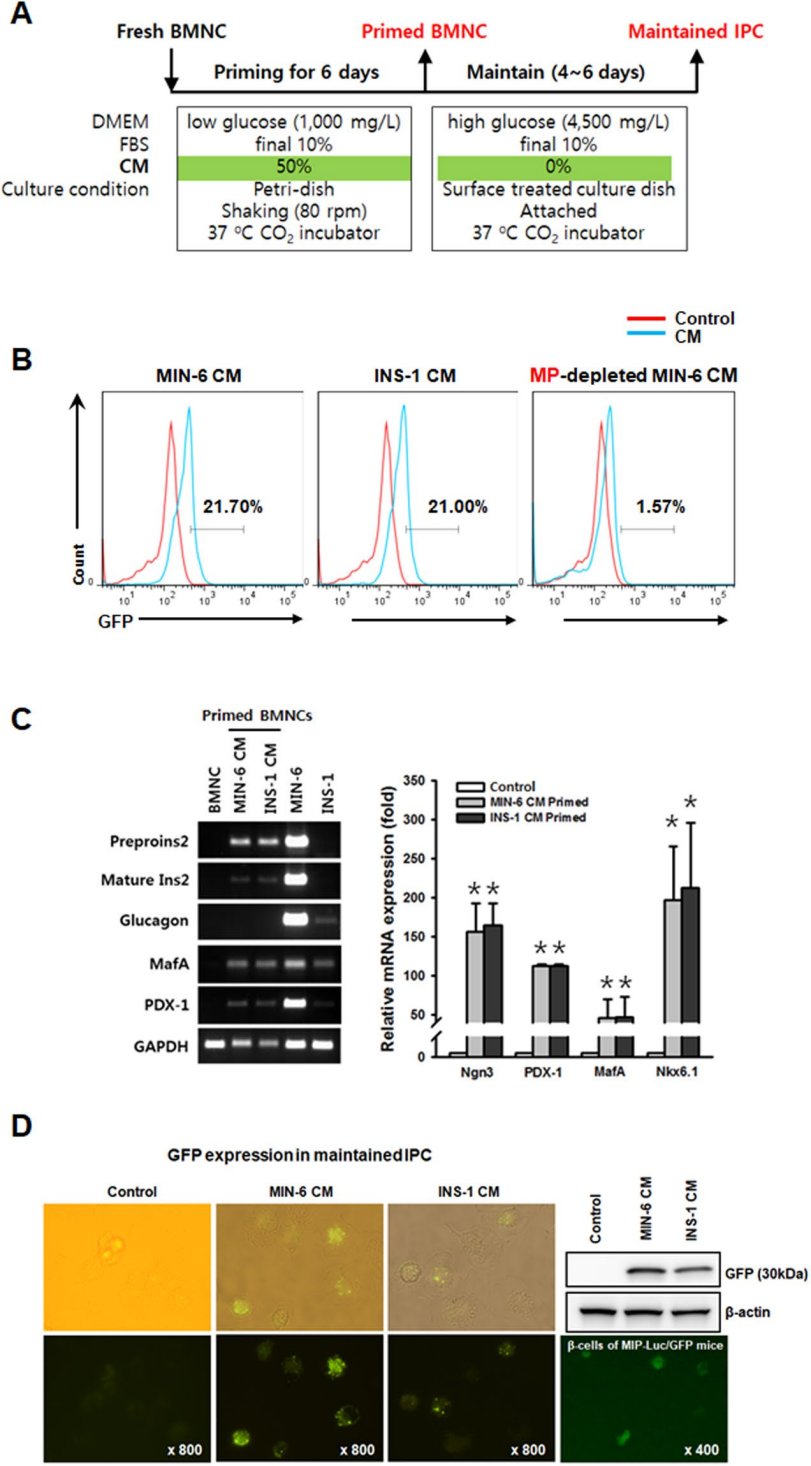
BMNCs differentiate into IPCs *in vitro*. (**A**) Outline of *in vitro* differentiation (priming + maintenance) protocol ruling out known differentiation inducing factors. (**B**) FACS analysis of BMNCs after 6 days of priming with MIN-6 CM (left), INS-1 CM (middle), and MPs-depleted MIN-6 CM (right). Representative histograms illustrating GFP expression as a marker of insulin promoter activation. Freshly prepared BMNCs of MIP-Luc/GFP mice were primed with differently prepared CM (blue line) for 6 days and GFP expression was compared to control BMNCs (red) in non-primed culture media. Priming with CM prepared from the supernatant of syngeneic β-cells (MIN-6) or xenogeneic β-cells (INS-1) directed GFP expression in BMNCs from MIP-Luc/GFP mice. BMNCs primed with MP-depleted MIN-6 CM do not express GFP as do control cells (red line). The text indicates the percentage of GFP expression compared to non-primed control cells. (**C**) Gene expression analysis of cells harvested at the end of 6 days of priming, compared with syngeneic (MIN-6 CM) or xenogeneic (INS-1 CM) β-cells as well as freshly isolated murine BMNCs (control). CM-primed BMNCs express β-cell markers (insulin 2, Ngn3, PDX-1, MafA, and Nkx6.1) but not glucagon. Freshly isolated BMNCs served as controls. Data are presented as mean ± S.E.M. of three independent experiments. *p < 0.05 for primed BMNCs versus. Freshly isolated BMNCs. (**D**) Primed BMNCs, which were further maintained for 6 days in the absence of CM still express GFP. Primed BMNCs were thoroughly washed with PBS and maintained for 6 more days under static conditions as described in (**A**). GFP expression under a fluorescent microscope is comparable to that of β-cells isolated from the pancreas of MIP-Luc/GFP mice and protein expression was confirmed by western blotting. Fluorescent images were obtained using a Nikon Eclipse Ti inverted fluorescent microscope.

We developed a simple and reproducible IPC-generating priming protocol (Fig. 2A) after extensive testing of various culture conditions. Priming with CM prepared from the culture supernatants of the syngeneic (MIN-6) or xenogeneic (INS-1) insulinoma cell lines directed the MIP-Luc/GFP derived BMNCs to express GFP within 6 days (Fig. 2B). However, microparticles (MPs)-depleted CM, in which large particles over 100 kDa have been removed, did not trigger GFP expression. This finding suggested that MPs shed from the β-cells under stress conditions were responsible for the differentiation of BMNCs into IPCs. Gene expression analysis of the primed BMNCs (Fig. 2C) revealed an increase in the mRNA expression of Ngn3, PDX-1, MafA, and Nkx6.1 which are known to directly regulate insulin transcription through the formation of a complex with transcriptional coactivators on the insulin promoter^37^. We observed similar priming effects with CM from the primary islets isolated from the xenogeneic rats (Fig. S2A). However, the BMNCs primed with normal culture media of MIN-6 (MIN-6 non-CM) or CM from a clonal endothelial bEND.3 did not express those β-cell specific genes (Fig. S2B). Primed BMNCs further maintained for 6 days in the absence of CM continued to express GFP (Fig. 2D).

### Pancreatic β-cell derived components induce the fate change of BMNCs into IPCs

**Figure 3:**
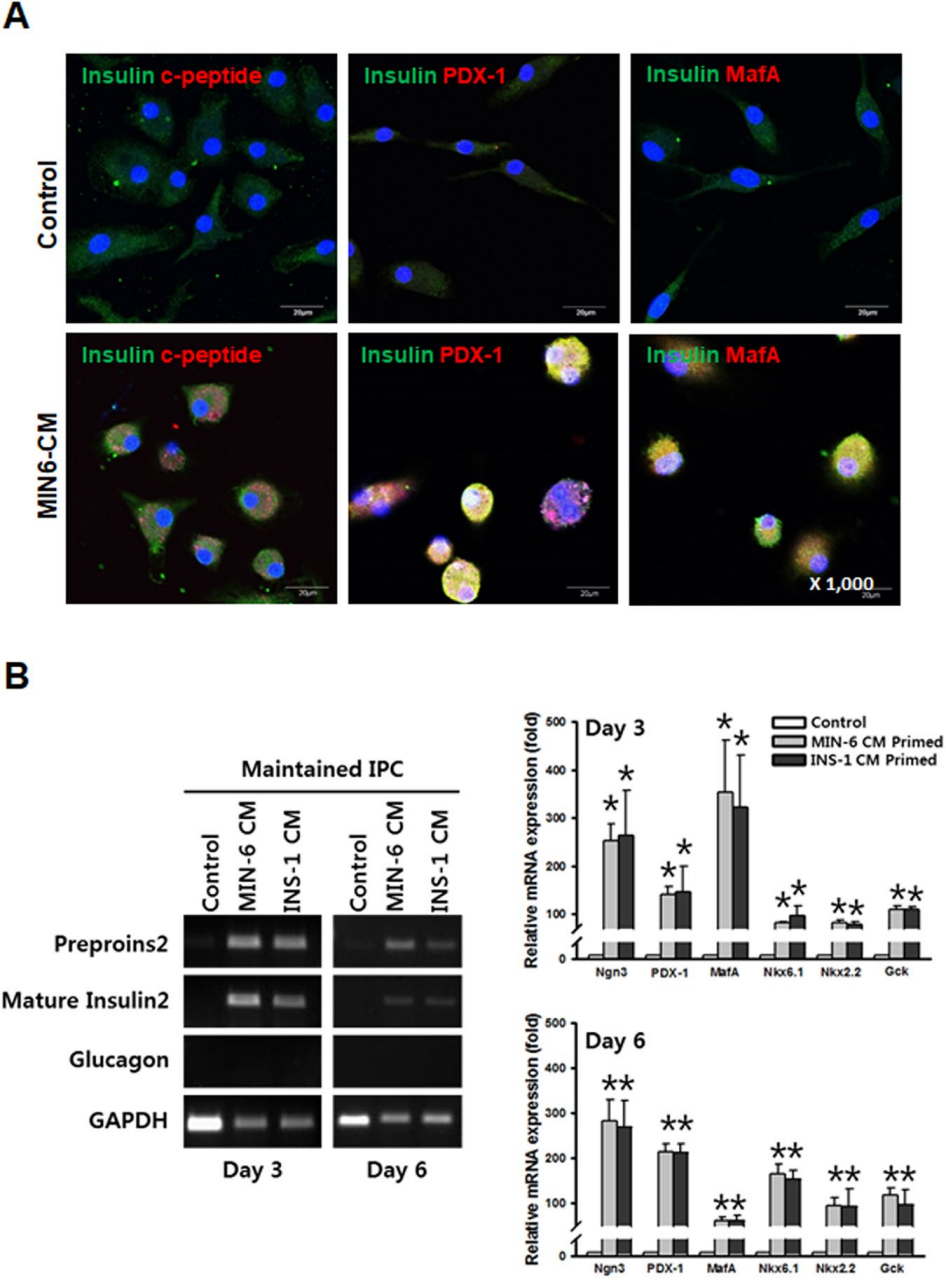
Primed BMNCs are further maintained in the absence of CM. Representative immunofluorescent staining of MIN-6 CM-primed BMNCs isolated from wild type C57BL/6 mice and comparable control cells at the end of maintenance (**A**). Both PDX-1 and MafA expression were found not only in the nucleus but also in the cytoplasm. Although the control cells were also weakly positive only for insulin staining, C-peptide, PDX-1, and MafA were not found in the control cells. Magnification 1,000×. Immunofluorescent staining for DAPI (blue), insulin (green) with C-peptide (red), PDX-1 (red), or MafA (red). Additional images are provided in Supplementary Fig. S3. All images were acquired with an Olympus FluoView^TM^ FV1000 confocal microscope. (**B**) Gene expression analysis of primed cells harvested after 3 and 6 days of maintenance in the absence of CM, compared with syngeneic (MIN-6) or xenogeneic (INS-1) β-cells as well as freshly isolated murine BMNCs. CM-primed BMNCs still expressed β-cell markers (insulin-2, Ngn3, PDX-1, MafA, Nkx6.1, Nkx2.2, and Gck) but not glucagon. Data are presented as mean ± S.E.M. of three independent experiments. *p < 0.05 for primed cells versus BMNCs.

BMNCs from the wild type C57BL/6 mice primed with MIN6-CM expressed the β-cell markers, including C-peptide, PDX-1, and MafA, within 6 days and could be maintained for an additional 6 days in the absence of CM (Figs 3A; S3). In contrast to this result, the expression of the β-cell markers in the BMNCs without CM from the insulinoma cell lines was negligible. Although the control cells were weakly stained for insulin, they were negative for C-peptide, PDX-1, and MafA suggesting a possibility of a nonspecific staining or contamination from insulin present in the culture medium serum (Fig. 3A). Gene expression analysis of the primed BMNCs maintained in the absence of CM (Fig. 3B) also indicated that insulin was produced by the primed BMNCs.

### Systemic infusion of primed BMNCs improves hyperglycemia in the diabetic mice

**Figure 4:**
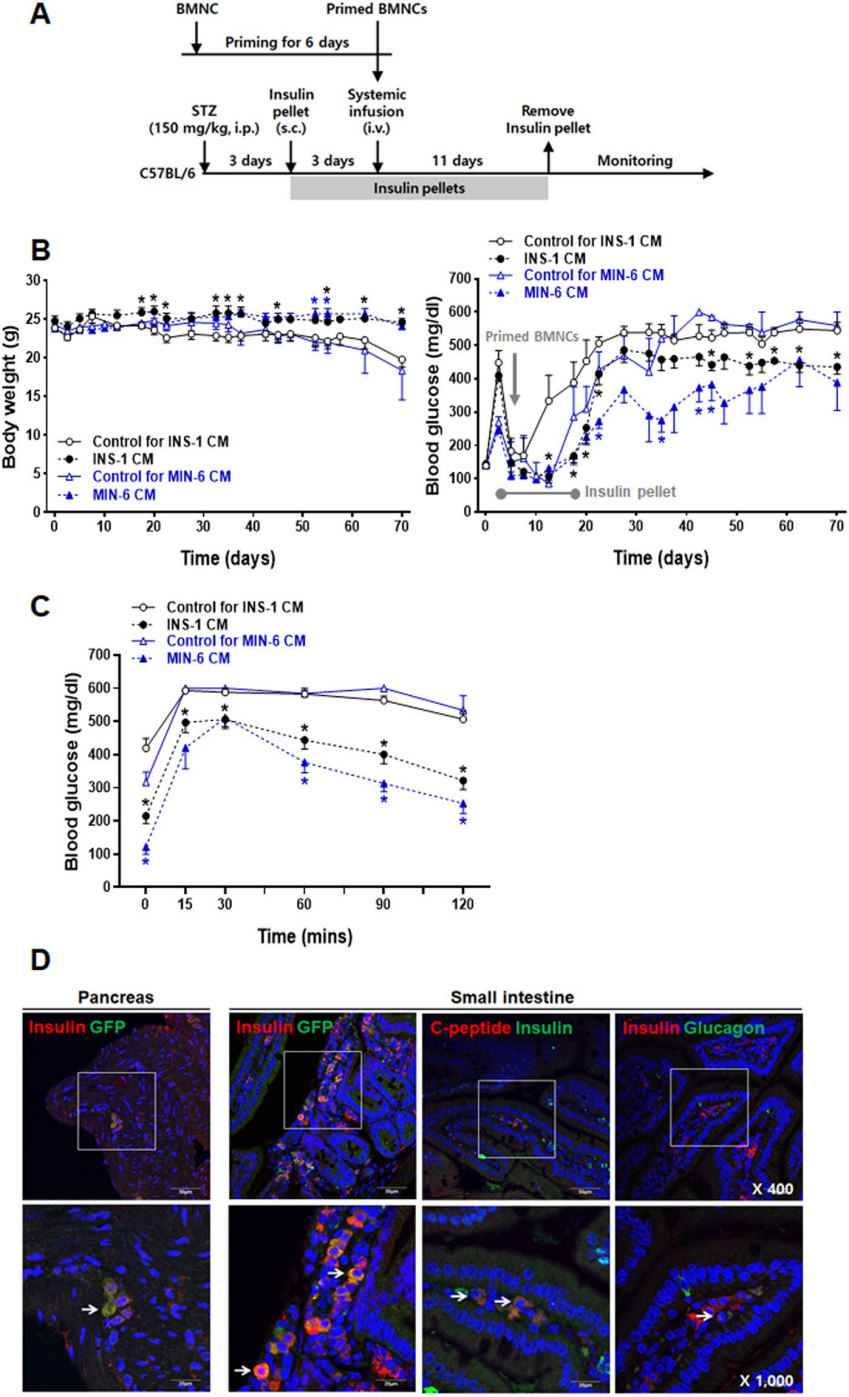
Systemic infusion of primed BMNCs improves hyperglycemia in STZ-induced diabetic mice. (**A**) Summarized scheme of animal experiments. Male C57BL/6 mice were injected with STZ (I.P.) and two insulin pellets were transplanted subcutaneously for 14 days beginning 3 days after the STZ injection, followed by monitoring for 70 days. After 6 days, primed MIP-Luc/GFP mouse BMNCs (3 × 10^6^ cells/mouse) were infused through the tail vein. (**B**) Body weight (left panel) and changes in random feeding blood glucose levels (right panel) of mice transplanted with primed cells (black filled circle for INS-1, n = 8; red filled triangle for MIN-6, n = 3) or comparable control cells (black empty circle for INS-1, n = 7; red empty triangle for INS-1, n = 3). (**C**) Blood glucose responses from the intraperitoneal glucose tolerance test (IPGTT). IPGTT (i.p. glucose (2 g/kg) injection) was performed after an overnight fast at 56 days after transplantation. Data are presented as mean ± S.E.M. *p < 0.05 for primed cells versus BMNCs (**D**) Representative immunofluorescent images of the pancreas and small intestines harvested 64 days after transplantation. Note the appearance of insulin and GFP double positive cells in the pancreas (arrow in the 1st panel from the left). Some of the cells present in the lamina propria of the intestinal villi are insulin/GFP (arrow in the 2nd panel from the left), or insulin and C-peptide double positive (arrow in the 3rd panel from the left), but insulin and glucagon double positive cells were rare (arrow in the 4th panel from the left). GFP positive cells were not found in the pancreatic or small intestine tissues from control mice. All images were acquired with an Olympus FluoView FV1000 confocal microscope. Scale bars in low magnification (50 μm, upper panels), in high magnification (20 μm, lower panel).

We tested whether primed BMNCs can regulate the blood glucose levels after systemic infusion of the primed BMNCs from the MIP-Luc/GFP mice into the diabetic mice through the tail vein (Fig. 4A). The baseline glucose levels of the STZ-induced diabetic mice were controlled by transplanting slow-release insulin pellets for 14 days. Systemically infused INS-1 CM-primed BMNCs, as well as MIN-6 CM-primed BMNCs, reproducibly protected the mice from a progressive decrease in body weight and maintained their blood glucose at lower levels compared with the corresponding levels in the diabetic mice transplanted with non-primed control BMNCs (Fig. 4B). Moreover, mice transplanted with the primed BMNCs showed significantly lower fasting blood glucose levels and improved glucose tolerance 56 days after transplantation compared to mice transplanted with non-primed BMNCs (Fig. 4C). Systemic infusion of the primed BMNCs also alleviated polyphagia and polydipsia frequently found in the diabetic mice (data not shown). We analyzed the pancreas and the intestinal tissues of the diabetic mice transplanted with INS-1 CM-primed BMNCs by immunofluorescence staining with antibodies against GFP and pancreatic α and β cell markers. We hardly observed GFP positive cells in the pancreas. Notably, few insulin single positive cells were found in the pancreas, indicating that residual or regenerated β cells could be presumed absent in the pancreatic tissues (Figs 4D; S4). On the other hand, we found a lot of GFP/insulin double positive cells scattered in the lamina propria of the intestinal villi, indicating that most transplanted cells moved to the intestine rather than pancreas. GFP/C-peptide double staining showed that insulin was properly processed in these cells (Figs 4D; S5A,B). Glucagon-expressing cells were rarely detected in the intestine, suggesting that the transplanted cells did not differentiate into α cells (Figs 4D; S5C). Although some populations of the intestinal cells are known to differentiate into IPCs^38–40^, GFP expression analysis revealed that transplanted cells, rather than endogenous cells, were the origin of the insulin and C-peptide double positive cells found in the intestine. However, we could not find any GFP-expressing cells in the pancreas or intestine of the diabetic mice transplanted with the control cells (data not shown).

## Discussion

In this study, we found that BMNCs can differentiate into IPCs *in vivo*, as demonstrated by the neogenesis of the GFP and insulin double positive cells in the STZ-treated MIP-Luc/GFP chimeric mice. This finding is in line with previous reports that showed the differentiation of BMNCs into functional cells in the injured tissues^24–26,41,42^. We also generated BMNCs-derived IPCs which express the β-cell-specific markers by *in vitro* priming of the freshly prepared BMNCs with CM prepared from the β-cell lines for 6 days. Furthermore, we demonstrated that transplantation of the *in vitro-*primed BMNCs contributed to blood glucose control in the diabetic mice and improved the fasting blood glucose levels and glucose tolerance, two months after the transplantation.

We established a simple and effective protocol for differentiation of IPCs from BMNCs, which has several advantages. First, freshly isolated BMNCs were utilized, unlike other protocols that use multiple stem cell sources manipulated in the culture dishes, including ESCs, induced pluripotent stem cells (iPCs), and adult stem cells, such as MSCs and HSCs. Additionally, BMNCs were easily primed with the CM prepared from the cultured β-cell lines without any chemical or cytokine supplements. Furthermore, only 6-day priming with CM was enough to differentiate the BMNCs into insulin-producing cells. We observed similar priming effects with the CM from the islets isolated from the xenogeneic rats but not with the CM from a cultured endothelial cell line. These results are in accordance with our previous report that liver conditioned media contribute to the differentiation of the bone marrow cells to hepatocytes^43^.

We assumed that β-cell-derived MP-associated factors were responsible for IPC differentiation, since MP-depleted CM did not trigger GFP expression in the BMNCs obtained from the MIP-Luc/GFP mice (Fig. 2B). Previously, we have shown that the use of the extracellular vesicle-mimetic nanovesicles derived from a mouse β-cell line can differentiate the bone marrow cells into IPCs *in vivo* and effectively regulate the blood glucose levels in the STZ-induced diabetic mice^44^. These results suggest that β-cell-derived factors in CM or MP might play an important role in the differentiation of IPCs from BMNCs.

More importantly, insulin-producing cells, insulin/GFP double positive cells as well as insulin single positive ones, were hardly found in the pancreas. It suggest that residual or regenerated β cells can be presumed absent in the pancreatic tissues, and the potential effect of resident regenerated cells is negligible. Interestingly, primed BMNCs infused via tail vein were found in the lamina propria of the intestinal villi, as demonstrated by the presence of the GFP/C-peptide and insulin/C-peptide double positive cells. The exact mechanism of engraftment of the transplanted primed BMNCs in the intestinal tissues is not known. However, we should note that diabetic cell therapy does not require functional integration of transplanted cells into pancreatic tissues because transplantation into ectopic sites can also provide an adequate insulin replacement to control hyperglycemia^45^.

An earlier study by Chen *et al*. reported that ectopic expression of Pdx1, MafA, and Neurog3 in the intestinal crypts results in their conversion into cells with β-like feature called “neoislets”, and importantly, intestinal neoislets are glucose-responsive and able to ameliorate hyperglycemia in STZ-induced diabetic mice. Talchai *et al*., based on the combination of genetic and cellular approaches of *Foxo1* ablation, concluded that somatic ablation of *Foxo1* in Neurog3-expressing (Neurog3^+^) enteroendocrine progenitors give rise to gut insulin-producing, glucose-responsive cells that express markers of mature β cells and secrete bioactive insulin as well as C-peptide. A growing literature thus demonstrates that IPCs can be produced from the gut or intestinal cells^38–40^, suggesting that the intestine is an accessible and abundant source of functional insulin-producing cells.

In the present study, we established a simple and effective protocol for differentiation of IPCs from BMNCs using β-cell-derived CM; however, CM or MPs are difficult to standardize. Moreover, MPs are able to mediate various unexpected pathophysiological fate changes in the stem cells^46^. Therefore, identification of the key components enclosed by or integrated into MPs shed from the β-cells might improve IPC differentiation efficiency by eliminating unwanted pathophysiological effects from other contaminated components. Additionally, identification of the components that direct the differentiation may enable us to generate BMNC-derived IPCs to treat DM more efficiently by minimizing the *ex vivo* manipulation requirements. Nevertheless, the methods that are effective in mice are not always effective in humans, and considerable research remains to be conducted to achieve the therapeutic goals for DM, although our novel approach to the generation of IPCs from BMNCs has potential for the development of a treatment for diabetic patients.

## Methods

### Animals

C57BL/6-Tg [(Ins2-luc/EGFP/TK) 300Kauf/J; Jackson Laboratories stock #012943, referred to as MIP-Luc/GFP in this study)] and wild type C57BL/6 mice (Orient-Bio Co. Ltd., Seoul, Korea) were maintained in our facility. Both strains were housed in a specific pathogen free zone under adequate temperature (23 ± 3 °C) and relative humidity (55 ± 5%) with a 12 h light/12 h dark cycle, and received autoclaved food and water *ad libitum*. All animal experiments were approved by the Animal Care and Use Committee (IACUC) of the Institute of Laboratory Animal Resources, Seoul National University, in accordance with the approved guidelines. MIP-Luc/GFP mice carried the tri-fusion transgene luciferase (Luc)/enhanced green fluorescent protein (EGFP)/thymidine kinase (TK) under the control of the mouse insulin-2 promoter. Transgene expression was determined by Luc enzymatic activity and GFP expression was exclusive to β-cells in the pancreatic islets. All efforts were made to minimize animal suffering as well as the number of animals used.

### Chimeric mice

Chimeric mice were generated by transferring BMNCs (1 × 10^6^ cells/mouse) from MIP-Luc/GFP mice into lethally (1,000 cGy) irradiated C57BL/6 mice (8–12 weeks old) via the tail vein. BMNCs were isolated from tibiae and femurs by flushing with Hank’s Balanced Salt Solution (HBSS). Chimeric mice were used for experiments 4 weeks after BM reconstitution.

### Cell sources

BMNC were prepared by flushing the femurs and tibias of MIP-Luc/GFP or wild type C57BL/6 mice. Whole BMNC were suspended in BD Pharm Lyse buffer (BD Biosciences, San Jose, CA) to remove RBCs, washed, and resuspended in the appropriate media for further experiments. MIN-6, a cloned pancreatic β-cell line from C57BL/6 mice, was cultured in DMEM medium (Hyclone #SH30243) supplemented with 10% fetal bovine serum (FBS, Gibco #26140-079), and 1% antibiotics (Gibco #15140122). INS-1, a cloned pancreatic β-cell line established from rats, was maintained in RPMI 1640 medium (Welgene #LM011-01) supplemented with 10% FBS and antibiotics. bEND.3, an endothelial cell line established from mice, was purchased from ATCC (#CRL-2299) and cultured in DMEM (Hyclone #SH30243) supplemented with 10% FBS and antibiotics.

### Diabetes induction

Diabetes was induced by a single I.P. injection of 150 mg/kg STZ (Sigma-Aldrich #S0130). Blood glucose levels in the mice were monitored using a standard glucometer (Accu-Chek Active; Roche Diagnostics, Mannheim, Germany) and blood from a tail pinprick. In some experiments, slow-release insulin pellets (LinBit; Linshin Canada; Toronto, ON, Canada) were implanted subcutaneously in the diabetic mice for 14 days beginning 3 days after the STZ injection to control the glucose levels.

### Intraperitoneal glucose tolerance test

Mice were fasted for 18 hrs and administered an I.P. injection glucose (2.0 g/kg body weight). Blood glucose levels were measured at the indicated times after glucose injection.

### Preparation of conditioned media

MIN-6 (1 × 10^7^), INS-1 (1 × 10^7^), and bEND.3 (5 × 10^6^) cells were briefly dispersed with complete media and attached for 24 hrs in a 100 mm cell culture dish. To expose cells to stress conditions, the culture media was exchanged with DMEM (Hyclone # SH30021) containing low glucose (1,000 mg/L) supplemented with 1% FBS and antibiotics and maintained for an additional 72 hrs in the same incubator. Culture supernatants were collected, centrifuged at 1,200 rpm for 5 min to eliminate the pellet, passed through a 0.45 μm syringe filter to obtain standard CM, and stored in aliquots at −80 °C for future use. CM from islets was prepared by same procedures from culture supernatant of isolated rat islets maintained in 2,000 IEQ/ml for 72 hrs in stress-inducing media, as previously mentioned. In some experiment MP-deprived CM was used instead. MP- depleted CM was prepared by removing large particles from the standard CM using centrifugal filter units (Amicon Ultra-4 Centrifugal Filter Unit with an Ultracel-100 membrane, Millipore #UFC810008).

### Transplantation of CM containing Matrigel

Matrigel grafts were prepared by mixing standard CM (100 μL) with the same volume of Matrigel (BD Matrigel Basement Membrane Matrix, BD Bioscience, San Jose, CA, USA), and transplanted into the subcutaneous space on the back of chimeric mice. Matrigel grafts were harvested on day 18 after transplantation, fixed, and embedded for immunofluorescence microscopic analysis.

### Systemic infusion of primed BMNCs in diabetic mice

Male C57BL/6 mice were injected with STZ (I.P.) and two insulin pellets were transplanted subcutaneously for 14 days beginning 3 days after the STZ injection, followed by monitoring for 70 days. After 6 days, primed BMNCs of the MIP-Luc/GFP mice (3 × 10^6^ cells/mouse) were infused to the STZ-treated mice through the tail vein.

### *In vitro* differentiation of murine BMNCs into IPCs

Whole BMNC were primed for 6 days in DMEM (Hyclone #SH30021) containing 10% FBS (final concentration) and 50% CM in suspension (primed BMNCs) in the absence of any supplements that are known to be essential to IPC differentiation. For characterization, primed BMNCs were washed twice with PBS and further maintained 3–6 days in DMEM (Hyclone #SH30243) containing 10% FBS and antibiotics under static condition (*in vitro* maintained BMNCs) as described in Fig. 2A.

### Reverse transcriptase polymerase chain reaction (RT-PCR) and quantitative real-time PCR (qPCR)

Total RNA was extracted from undifferentiated BMNCs and from primed BMNCs using the TRIzol Reagent (Invitrogen). Total RNA (500 µg) was reverse transcribed into first-strand cDNA using SuperScript II Reverse Transcriptase (Promega) according to the manufacturer’s instructions. RT-PCR was performed to amplify each cDNA using the AccuPower PCR premix (Promega). PCR products were separated on 1.5% agarose gel and stained with Dyne LoadingSTAR (DYNEBIO, A750). Q-PCR was performed using SYBR Premix Ex Taq (Takara, Shiga, Japan) and a LightCycler 96 (Light Cycler 96; Roche, Mannheim, Germany). The hypoxanthine phosphoribosyl transferase (HPRT) mRNA level was used as the internal control. Relative expression levels were calculated using the 2^−ΔΔCt^ method after normalization to β-actin. The primer sequences are shown in Supplementary Table 3.

### Flow cytometry

Prepared cells were washed twice in staining buffer (HBSS containing 2% FBS). To quantify the GFP expressing cells controlled by insulin-2 promoter activation, primed BMNC were washed twice with PBS and incubated with the specific antibodies (CD45-APC, eBioscience #17-0451) for 30 min at 4 °C. Flow cytometry was performed using a BD LSR II instrument (Becton Dickinson) with subsequent analysis using the FlowJo data analysis software (FlowJo, Ashland, OR) with at least 50,000 events being acquired.

### Measurement of secreted insulin

The culture media from primed cells was replaced with high glucose containing DMEM (Hyclone #SH30243) supplemented with 10% FBS and antibiotics. After 3 days, the supernatants were collected and insulin levels were measured using an insulin immunoassay kit (ALPCO, Salem, NH, USA) according to the manufacturer’s instructions.

### Immunofluorescent staining

Sectioned tissues or paraformaldehyde fixed cells were washed three times with PBS, then blocked with PBS containing 5% normal goat serum (NGS) and 0.3% Triton X-100 for 15 min. After blocking, the cells were incubated with primary antibodies (Supplementary Table 2) in 2.5% NGS overnight at 4 °C. The next day, the cells were washed three times with PBS-Tween 20 (PBS-T, 0.01%), and then incubated with secondary antibodies in 2.5% NGS for 1 hr at room temperature. Cells were counterstained with 4,6-diamidino-2-phenyldole (DAPI; 1:2,500; Molecular Probe), and an Olympus FluoView FV1000 confocal microscope fitted with the appropriate filters was used to obtain the images.

### Western blot analysis

Cell lysates were prepared in lysis buffer containing 20 mM Tris-HCl (pH 7.4), 1% NP-40, 10 mM Na_4_P_2_O_7_, 5 mM EDTA, 100 mM NaF, 2 mM Na_3_VO_4_, 7 μg/mL leupeptin, 7 μg/mL aprotinin, and 1 mM phenylmethylsulfonyl fluoride. Whole-cell lysates were subjected to sodium dodecyl sulfate-polyacrylamide gel electrophoresis (SDS-PAGE). Proteins on the gel were transferred onto a nitrocellulose membrane. The membranes were incubated in blocking solution (5% skim milk) and then with specific primary antibodies in 0.1% Tween® 20-Tris-buffered saline. Hybridized primary antibodies were detected using a horseradish peroxidase-conjugated IgG antibody (Santa Cruz Biotechnology). Bands were detected using an enhanced chemiluminescence kit (Thermo, Rockford, IL, USA).

### Statistical analysis

Statistical analysis was performed with the Statistical Analysis System (SAS) software package (version 9.3; SAS Institute, Cary, NC). Insulin secretion and gene expression were analyzed by non-parametric repeated measures ANOVA. Changes in body weight and blood glucose levels, including IPGTT, were analyzed for each cage by mixed effects regression models that fitted random effects for animals and fixed effects for time, group and an interaction of time. If the interaction was not-significant, the interaction term was removed and the model was rerun. Bonferroni corrections were used to adjust p-values for multiple testing. Two-sided p-values of < 0.05 were considered statistically significant. Data were expressed as mean ± S.E.M.

## Supplementary information

Direct differentiation of bone marrow mononucleated cells into insulin producing cells using pancreatic β-cell-derived components.
