# Supplementary material for: Direct differentiation of bone marrow mononucleated cells into insulin producing cells using pancreatic β-cell-derived components
Source: Sci Rep. 2019 Mar 29;9:5343. doi: 10.1038/s41598-019-41823-9 (PMC6441031; doi:10.1038/s41598-019-41823-9)
Supplement: Supplementary file 1 — Direct differentiation of bone marrow mononucleated cells into insulin producing cells using pancreatic β-cell-derived components. [file 41598_2019_41823_MOESM1_ESM.docx]

**Supplementary Information for:**

**Direct differentiation of bone marrow mononuclear cells into insulin producing cells using pancreatic β-cell-derived components.**

**Authors**

Ju Eun Oh^1,2^, Ok Kyung Choi^1^, Ho Seon Park^1^, Hye Seung Jung^1,4^, Su Jeong Ryu^3^, Yong Deok Lee^1^, Seung-Ah Lee^2^, Sung Soo Chung^1^, Eun Young Choi^3^, Dong-Sup Lee^3^, Yong Song Gho^5^, Hakmo Lee^1,6,*^, Kyong Soo Park^1,2,4,*^.

**Author affiliations**

^1^Biomedical Research Institute, Seoul National University Hospital, Seoul 03080, Republic of Korea.

^2^Department of Molecular Medicine and Biopharmaceutical Sciences, Graduate School of Convergence Science and Technology, Seoul National University, Seoul 03080, Republic of Korea.

^3^Department of Biomedical Sciences, Seoul National University College of Medicine, Seoul 03080, Republic of Korea.

^4^Department of Internal Medicine, Seoul National University College of Medicine, Seoul 03080, Republic of Korea.

^5^Department of Life Sciences, Pohang University of Science and Technology, Pohang, Gyeongbuk 37673, Republic of Korea.

^6^ Veterans Medical Research Institute, Veterans Health Service Medical Center, Seoul 05368, Republic of Korea.

**Supplementary Methods**

**Rat islet isolation.** Rat islets were isolated from Sprague Dawley (SD) rats (8-12 weeks), which were purchased from Orient-Bio (Korea), as described previously^1^.

**Bioluminescence imaging.** *In vivo* bioluminescence imaging was performed using an IVIS 100 imaging system with a charge-coupled device (CCD) camera (Caliper Life Science, Waltham, MA, USA), as previously described^2^. Mice on the imaging stage were given an I.P. injection of the D-luciferin substrate (150 mg/kg body weight; Molecular Probes, Eugene, Oregon, USA) under anesthesia using 1.5% isoflurane gas in oxygen. Five minutes later, the mice were sacrificed and positioned supine to image the ventral surface to reveal the organ. Relative intensities of emitted light were presented as pseudo color images ranging from red (most intense) to blue (least intense), which were superimposed on gray-scale photographs using the Living Image (ver. 2.12; Xenogen, Alameda, CA, USA) and IGOR (WaveMetrics, Portland, OR, USA) image analysis software packages. Signal intensities emitted by the region of interest (ROI) were measured and expressed as photon fluxes (photon s^−1^cm^−1^sr^−1^), which refer to the photons emitted from a solid angle of a sphere. The instrument background was subtracted electronically, both from the images and from the measurements of photon flux.

**Supplementary Figure 1.**


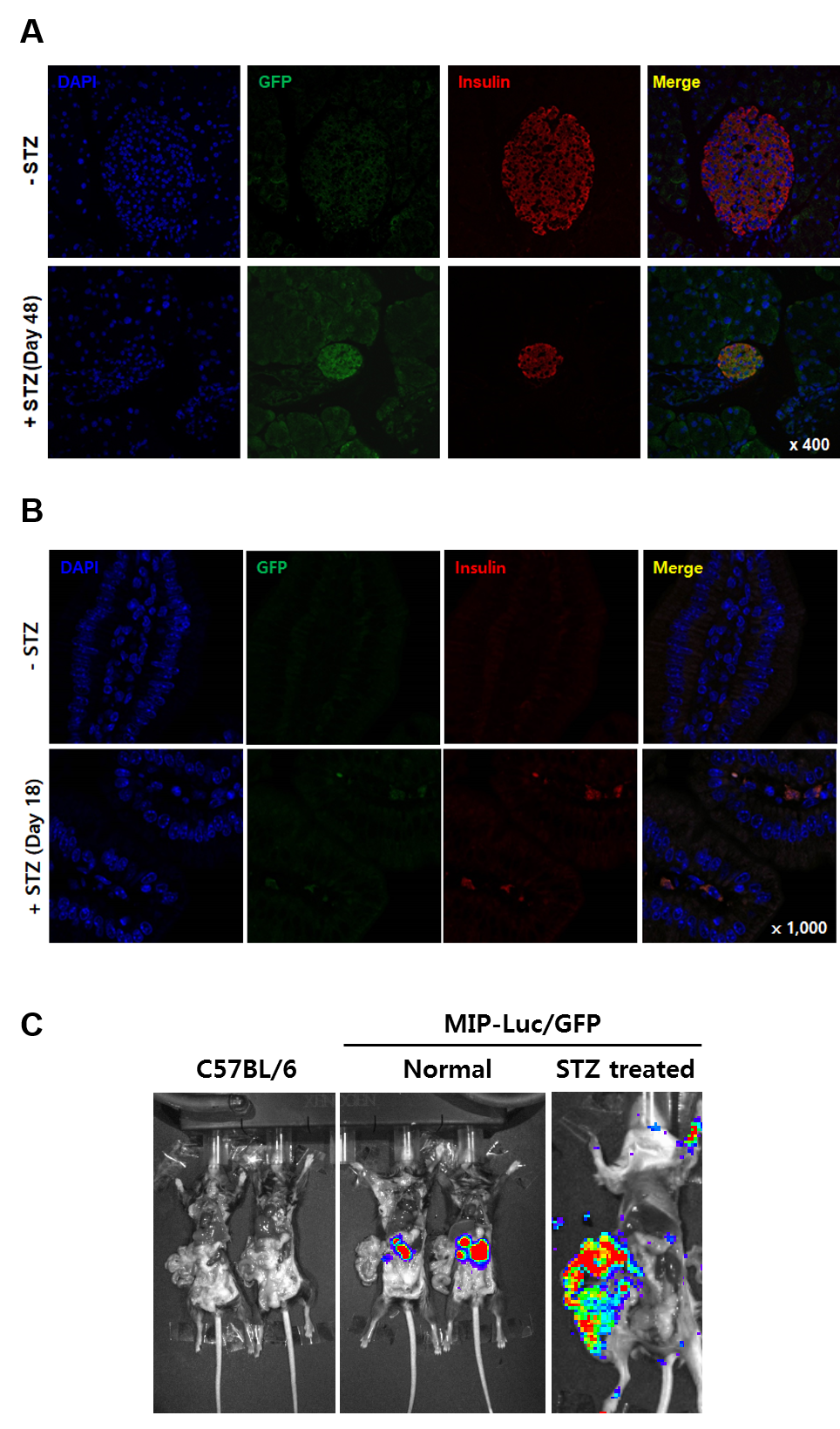


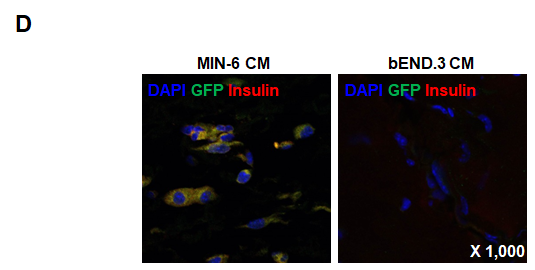


**Supplementary Figure 1. The contribution of BMNCs to the neogenesis of IPCs.** (A) Immunofluorescent staining of pancreatic tissues harvested from chimeric C57BL/6 mice bearing BMNCs from MIP-Luc/GFP mice. Each horizontal panel show representative staining of the pancreas harvested from a control mouse (- STZ) and a diabetic (48 days after STZ injection showing hyperglycemia, + STZ). Images were acquired with an Olympus FluoView FV1000 confocal microscope. Magnification 400×. (B) Immunofluorescent staining of small intestine tissues from control (vehicle treated, - STZ) or diabetic (STZ-treated, + STZ)-MIP-Luc/GFP mice on day 18. Note the appearance of insulin and GFP double positive cells in the lamina propria of the intestinal villi of diabetic mice (lower panel) but the absence of those cells in control mice (upper panel). Images were taken with an Olympus FluoView FV1000 confocal microscope. Magnification 1,000×. (C) Live imaging of luciferase signaling in wild type C57BL/6 (left), normal (middle)- and STZ treated (right)-MIP-Luc/GFP mice. Note that the luciferase signaling shifted from the pancreas to the intestinal regions in diabetic MIP-Luc/GFP mice on day 18 after STZ treatment. (D) Representative immunofluorescent staining of a Matrigel graft harvested 18 days after transplantation as described in the supplementary methods. GFP (green) and insulin (red) double-positive cells are present in the Matrigel graft containing CM of MIN-6 (left panel) but not in the bEND.3 CM (right panel). Magnification 1,000×. Image was acquired with an Olympus FluoView FV1000 confocal microscope.

**Supplementary Figure 2.**


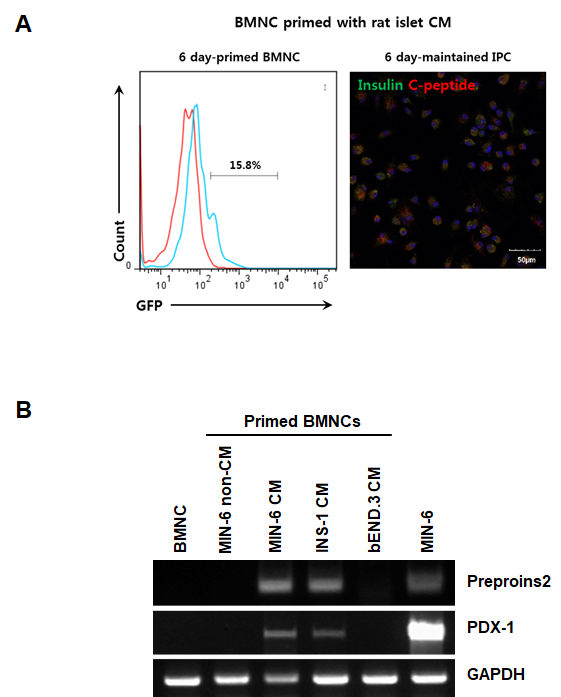


**Supplementary Figure 2.** (A) Differentiation of rat islet-derived CM-primed MIP-Luc/GFP mouse derived- BMNCs (left, 6 day-primed BMNC) or wild type C57BL/6 mouse derived-BMNCs (6 day-primed BMNCs further maintained for 6 days in the absence of CM) into IPCs. Fluorescent images were obtained using Nikon Eclipse Ti inverted fluorescent microscope. Scale bar 50 μm). (B) Gene expression analysis of primed BMNCs harvested at 6 days reveals that β-cell specific genes are not expressed in BMNCs primed with MIN-6 non-CM (culture media of MIN-6 under non-stress condition) and with bEND.3 CM. CM, conditioned media; non-CM, culture media under non-stress condition.

**Supplementary Figure 3.**


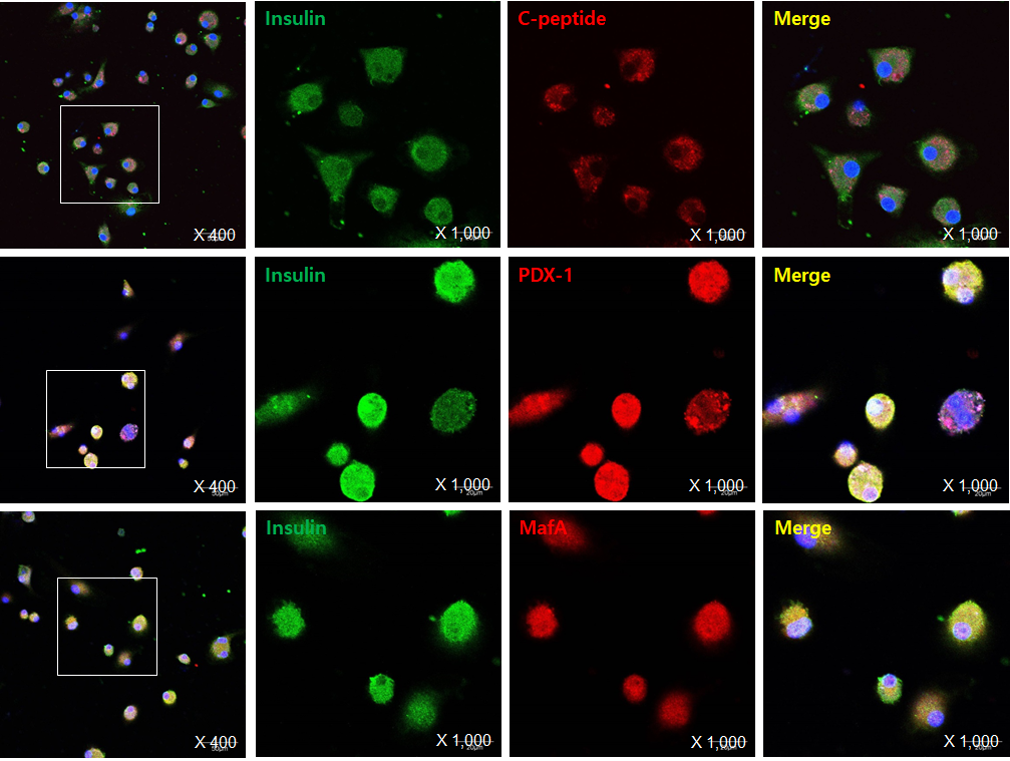


**Supplementary Figure 3. Immunofluorescent staining of MIN-6 CM-primed BMNCs maintained for an additional 6 days in the absence of CM.** Images were acquired with an Olympus FluoView FV1000 confocal microscope. Scale bars, 50 μm (low magnification, X400) and 20 μm (high magnification, X1,000).

**Supplementary Figure 4.**


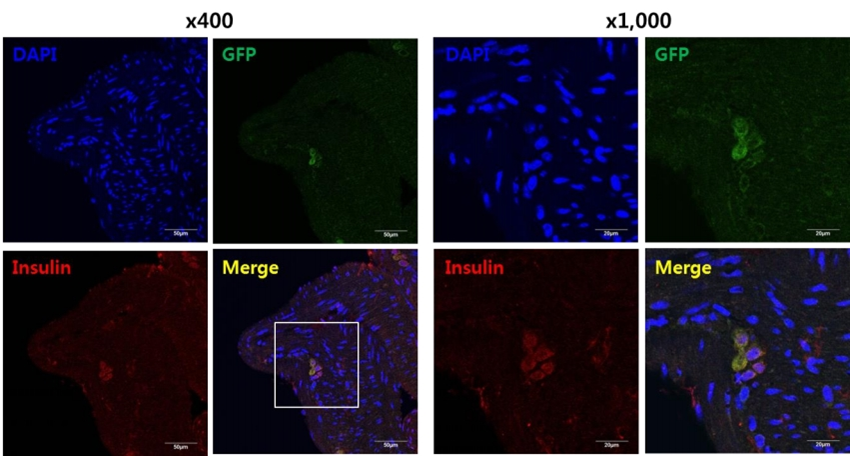


**Supplementary Figure 4. Single and merged immunofluorescent images of pancreas harvested at 64 days after primed BMNC transplantation.** Note the appearance of insulin and GFP double positive cells. Images were acquired with an Olympus FluoView^TM^ FV1000 confocal microscope. Scale bar, 50 μm (low magnification) and 20 μm (high magnification).

**Supplementary Figure 5.**


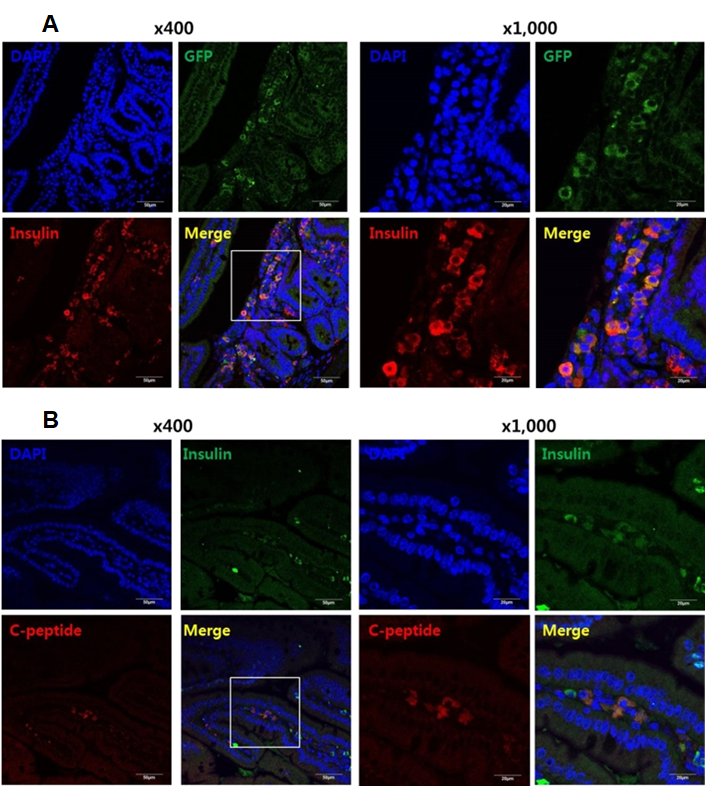


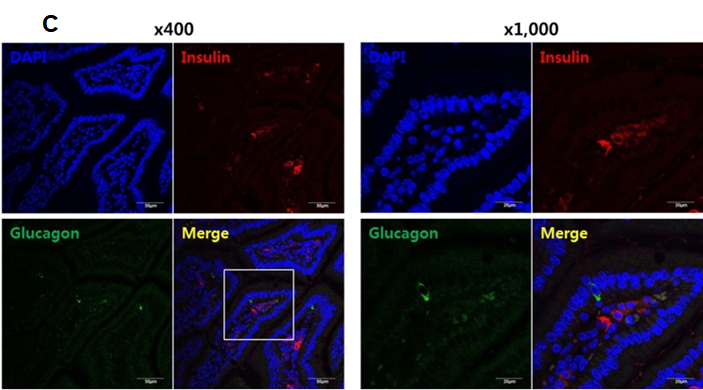


**Supplementary Figure 5. Single and merged immunofluorescent images of the small intestine harvested 64 days after transplantation of primed BMNCs.** Note the appearance of insulin (red) and GFP (green) (A), insulin (green) and C-peptide (red) (B), and insulin (red) and glucagon (green) (C) double positive cells. Images were acquired with an Olympus FluoView^TM^ FV1000 confocal microscope. Scale bars, 50 μm (low magnification) and 20 μm (high magnification).

**Supplementary Table 1. Quantification of GFP-expressing cells present in normal or diabetic chimeric mice.**

|  |  | **Blood glucose (mg/dl)** | **Number of cells counted** | | |
| --- | --- | --- | --- | --- | --- |
|  |  |  | **GFP/Insulin** | **GFP/PDX-1** | **GFP/Glucagon** |
| **No STZ** | Normal | 133 | ND^a^ | ND | ND |
| **STZ**  **(150 mg/kg, I.P.)** | Day 24 | 497 | 31/37  (8)^b^ | 286/663  (9) | ND |
|  | Day 34 | 422 | 161/169  (10) | 358/463  (9) | ND |
|  | Day 48 | 168 | 2757/2769  (19) | 2139/2223  (17) | ND |

^a^, ND, Not detected

^b^, Number of islets counted

**Supplementary Table 2. List of antibodies used for immunofluorescent staining.**

|  | **Antibodies** | **Catalog #** | **Dilution** |
| --- | --- | --- | --- |
| **Unconjugated primary antibodies** | Insulin | Sigma (#I-2068) | 1:100 |
|  | Insulin | Cell Signaling (#3014) | 1:100 |
|  | C-peptide | Cell Signaling (#4593) | 1:100 |
|  | GFP | Abcam (#ab6556) | 1:100 |
|  | Glucagon | Sigma (#G-2654) | 1:1000 |
|  | PDX-1 | R&D (#MAB2419) | 1:100 |
|  | PDX-1 | Abcam (#ab98298) | 1:100 |
|  | MafA | Abcam (#ab17976) | 1:100 |
| **Conjugated secondary antibodies** | Alexa Fluor 594 anti-mouse IgG | Invitrogen (#A11005) | 1:1000 |
|  | Alexa Fluor 488 anti-mouse IgG | Invitrogen (#A11001) | 1:1000 |
|  | Alexa Fluor 594 anti-rabbit IgG | Invitrogen (#A11012) | 1:1000 |
|  | Alexa Fluor 594 anti-guinea pig IgG | Invitrogen (#A11076) | 1:1000 |

**Supplementary Table 3. List of primers used for PCR analysis.**

|  | **Forward Primers** | **Reverse Primers** |
| --- | --- | --- |
| **Preproinsulin 2** | ATGTGTCTTTGCTTCTGTGCTG | ATCTACAATGCCACGCTTCTG |
| **Mature insulin 2** | CTGCTCTTCCTCTGGGAGTC | AGCTCCAGTTGTGCCACTTGT |
| **Glucagon** | TGAAGACAAACGCCACTCAC | CAGCATGCCTCTCAAATTCA |
| **Neurogenin 3 (Ngn3)** | AGCGGACCACAGCTTCTATG | CAGGGAATTCCTCCAATGAG |
| **PDX-1** | AGGTGCTTACACAGCGGAAC | TTCAACATCACTGCCAGCTC |
| **MafA** | GAGGTCATCCGACTGAAACAGAAG | ACTTCTCGCTCTCCAGAATGTGC |
| **Nkx6.1** | GGAAGAGAAAACACACCAGACC | ACCAGACCTTGACCTGACTCTC |
| **Nkx2.2** | TTATGGCCATGTACACGTTCTG | ACATAACCCACCGAAAACAAAC |
| **Glucokinase (Gck)** | GAAGGAAAAGGTAGAGCAGATCC | GTAGGTGGGCAACATCTTTACAC |
| **GAPDH** | AGGTCGGTGTGAACGGATTTG | TGTAGACCATGTAGTTGAGGTCA |
| **HPRT** | TGGATATGCCCTTGACTATAATGAGTACTTCAG | GTCTGGGGACGCAGCAACTGAC |

**Supplementary References**

1. Lee, H., *et al*. 4-Deoxypyridoxine improves the viability of isolated pancreatic islets ex vivo. *Islets* **5**, 116-121 (2013).

2. Ryu, S.J., *et al.* Alleviation of skin inflammation after Lin(-) cell transplantation correlates with their differentiation into myeloid-derived suppressor cells. *Sci Rep* **5**, 14663 (2015).
